# Social determinants and distance from certified treatment centers are associated with initiation of esketamine nasal spray among patients with challenging-to-treat major depressive disorder

**DOI:** 10.1097/MD.0000000000032895

**Published:** 2023-02-17

**Authors:** Joshua Liberman, Jacqueline Pesa, Pinyao Rui, Kruti Joshi, Lisa Harding

**Affiliations:** a Health Analytics, LLC, Columbia, MD; b Janssen Scientific Affairs, Titusville, NJ; c Depression MD, Milford, CT.

**Keywords:** health care access, major depressive disorder, social determinants of health

## Abstract

Indicated for treatment-resistant depression or major depression with suicidal ideation, esketamine (ESK) is self-administered under supervision at certified treatment centers. Our study was to determine if social determinants of health and distance were associated with ESK utilization. We conducted a retrospective cohort study among 308 US adults initiating ESK between October 11, 2019 and December 31, 2020 and 1540 propensity-score matched controls with treatment-resistant depression or major depression with suicidal ideation. Adjusting for demographics, prior health care utilization and comorbidities, social determinant variables and distance were regressed separately on each outcome: ESK initiation, failure to complete induction (8 treatments in 45 days), and discontinuation within 6 months. ESK initiation was associated with higher population density (odds ratio [OR]: 2.12), American Indian, Alaska Native, Native Hawaiian, Other Pacific Islander (OR: 3.19), and mental health (OR: 1.55) and primary care providers (OR: 1.55) per capita. Lower likelihood of ESK initiation was associated with living > 7.2 miles from a treatment center (OR: 0.75), living in rural areas (OR: 0.64), and percent non-Hispanic African American (OR: 0.58) and Hispanic (OR: 0.40). Health care providers should tailor patient engagement strategies to mitigate potential barriers to initiating and continuing appropriate treatment. Failing to complete induction was associated with substance use disorder and longer distance to treatment center was associated with discontinuation (hazard ratio: 1.48), as was percent Asian population (hazard ratio: 1.37). Prior psychiatric care and residence in counties with high rates of primary care providers per capita, unemployment, and high school graduation were associated with both higher likelihood of completing induction and lower likelihood of discontinuation.

## 1. Introduction

Major depressive disorder (MDD) is a chronic, episodic condition that can adversely affect the ability to function, to work, to participate in school, and to enjoy life.^[1–4]^ Individuals with MDD are also at elevated risk for suicidal thoughts and behaviors.^[5]^ Despite numerous therapeutic options, inadequate or incomplete response to treatment is common^[6–8]^ and an estimated 30% of individuals with MDD are classified as treatment resistant, indicating ≥ 2 failed trials of antidepressant therapy of adequate dose and duration.^[9]^

Lack of equitable access to mental health care services is well documented and a significant challenge. Non-White racial and Hispanic ethnic groups use significantly less mental health care,^[10–12]^ even among individuals with severe mental illness.^[13]^ Lower socioeconomic status is associated with elevated mental health visit no-show rates,^[14,15]^ and individuals with limited proficiency with the English language use fewer services.^[16]^ Individuals with fewer economic resources are more likely to report lack of transportation as a barrier to seeking care,^[17]^ which is consistent with a growing body of evidence documenting an inverse association between distance to health care providers and utilization.^[18,19]^

The insights above imply patient-level decision-making. However, research also indicates that the uptake of novel drug therapies may be associated with physician-level decision-making. A systematic literature review conducted by Lublóy^[20]^ reported that prescribers were more likely to recommend novel drug therapies for patients with higher income levels or with private insurance and were less likely to recommend novel drug therapies for African American race or Hispanic ethnicity patients.

Esketamine (ESK), in conjunction with an oral antidepressant, is indicated for adults with treatment-resistant depression (TRD) or with MDD associated with acute suicidal ideation or behavior (MDSI).^[21]^ Because of the risks of serious adverse outcomes resulting from sedation and dissociation and of the risk of abuse and misuse, the approved treatment regimen requires self-administration in a certified treatment center under the direct observation of a health care provider. ESK treatment begins with induction, requiring twice weekly visits for 4 weeks. After induction, TRD patients (but not always MDSI patients) transition to a maintenance phase, with weekly administration for 4 weeks followed by biweekly administration. The requirement for repeated in-person visits places a high demand on the patient and creates a unique opportunity to evaluate factors associated with initiating and continuing treatment. The objective of this research was to determine if social determinants of health (SDoH) and distance to be traveled contributed to the initiation and continuation of ESK treatment.

## 2. Methods

### 2.1. Study design

This is a retrospective observational comparative study among US adults who initiated ESK at a certified treatment center between October 11, 2019, and December 31, 2020, (ESK initiators) and a propensity-score matched population of controls eligible for, but who did not initiate, ESK treatment. The study protocol was approved by the Advarra institutional review board.

### 2.2. Data source(s)

Study participants were identified using a commercially available administrative medical and pharmacy claims database, licensed from Clarivate™ (London, United Kingdom). SDoH were measured at the county level from the area health resources file (AHRF) available from the Department of Health and Human Services (www.data.hrsa.gov/topics/health-workforce/ahrf).

### 2.3. Identification and selection of study participants

To be eligible for inclusion in this study as an ESK initiator, an individual must have had ≥ 1 claims for ESK between October 11, 2019, and December 31, 2020, be 18 years of age or older as of ESK initiation date (index), and have evidence of continuous insurance coverage – measured by a medical or pharmacy claim in each 3-month calendar quarter of the 12-month period prior to index (baseline) and 6-month period following initiation (follow-up). Individuals were excluded from the ESK initiator group if they had 2 medical claims, ≥30 days apart, for an outpatient service associated with a diagnosis of bipolar disorder (international classification of diseases, 10th edition (ICD-10)-CM: F31.x), schizophrenia (ICD-10-CM: F20.x), or schizoaffective disorder (ICD-10-CM: F25.x) during the 18-month study period. Two individuals were excluded based on excessive distance traveled (83 miles and 103 miles) to receive ESK treatments.

To be eligible for inclusion in the study, a control: met criteria for MDD defined by ≥ 2 medical claims, 30 days apart, with ICD-10-CM F32.X (excluding F32.8), F33.X (excluding F33.8) between October 11, 2017, and December 31, 2020; had ≥ 1 medical claim, including a diagnosis of MDD, or 1 antidepressant medication claim on or after October 11, 2019; resided in a state with ≥ 1 certified treatment center; resided within 64 miles of a certified treatment center; be 18 years of age or older as of index (see below); have evidence of continuous insurance coverage – measured by a medical or pharmacy claim in each 3-month calendar quarter of the 12-month baseline and 6-month follow-up periods; and met criteria for either TRD or MDSI. To be defined with TRD, a control had to initiate a third antidepressant treatment course (index) between October 11, 2019, and December 31, 2020, following 2 failed treatment courses of adequate dose and duration as defined by the American Psychiatric Association.^[22]^ A treatment course included antidepressant monotherapy or antidepressant therapy augmented with another antidepressant. A treatment course was considered to have failed, if any of the following occurred: discontinuation (>30 days without medication); augmentation with an antipsychotic medication; or switching to another antidepressant medication or antipsychotic medication.^[23]^ To be defined as MDSI, an individual had ≥ 1 medical claim associated with suicidal ideation or suicide attempt between October 11, 2017, and December 31, 2020, (see Table S1, Supplemental Digital Content, http://links.lww.com/MD/I441, that lists ICD-10 codes for suicidal behaviors). For the MDSI population, index is the earliest date associated with the initiation of an antidepressant treatment following the MDSI diagnosis and occurring between October 11, 2019, and December 31, 2020. Individuals were excluded from the control group if they had a claim for ESK administration or had 2 medical claims, at least 30 days apart, for an outpatient service associated with a diagnosis of bipolar disorder (ICD-10-CM: F31.x), schizophrenia (ICD-10-CM: F20.x), or schizoaffective disorder (ICD-10-CM: F25.x) during the study period (see Figure S1, Supplemental Digital Content, http://links.lww.com/MD/I442, flow diagram for the identification and selection of eligible individuals).

### 2.4. Matching

ESK initiators and controls were matched using a propensity score at a 1:5 ratio with the greedy matching approach and calipers of width equal to 0.02. The propensity score was derived from the baseline period (12 months prior to index) and included age, sex, insurance (Medicaid, Medicare, commercial, or Veterans affairs/other), select medical and psychiatric comorbidities, Charlson comorbidity index (CCI), and measures of all-cause and psychiatric health care utilization including office visits (overall and for primary care and psychiatry, separately), psychotherapy sessions, hospital-based outpatient visits, other outpatient visits, mental health facility visits, emergency department visits, and inpatient admissions.

### 2.5. Outcome and predictor variables

The 6-month period following index (follow-up) was used to assess the outcomes of completion of the ESK induction phase as we all as discontinuation of treatments among the ESK initiator group only. ESK use was defined by a paid medical or pharmacy claim for ESK (health care common procedure coding system codes G2082, G2083, S0013, and XW097M5; national drug codes 50458002800, 50458002802, and 50458002803). The study included 3 measures of ESK utilization: initiation; completion of the induction phase within 45 days of initiation; and discontinuation, measured as a 60-day period without administration. Recommended dose and administration regimen vary by indication. For individuals with MDSI, 2 administrations per week for 4 weeks at a dose of 84 mg. For individuals with TRD, 2 administrations per week for 4 weeks beginning with a 56-mg dose with potential escalation to 84 mg if deemed medically appropriate, followed by 1 administration per week for Weeks 5 through 8, and then every 2 weeks from that point forward. Completion of the induction phase was measured among all ESK initiators and discontinuation was measured only among initiators with no evidence of MDSI.

Distance was measured as the geodesic distance (in miles) from the centroid of the assigned residential zip code and the address of active risk evaluation and mitigation strategy-certified treatment centers, using the longitude and latitude of each. As the dataset included only the first 3 digits of the patient zip code, the provider zip code served as a proxy for patient residence. The provider zip code was assigned hierarchically in this order: 3-digit patient zip code matches first 3 digits of provider zip code; if multiple matching 5-digit zip codes of providers, the zip code belonging to the primary care provider (PCP) with the highest number of encounters; if no matching PCP, the following sequence of providers were used and the process repeated: primary care clinic, psychiatry care, therapeutic care, and other professional care (e.g., outpatient facility and inpatient facility). Treatment center activation and opt-out dates were used to select only active treatment centers for the duration of each patients follow-up period. For ESK initiators, distance was estimated from the assigned residential zip code of the patient to the ESK treatment center associated with the first (index) ESK claim. For controls, distance was estimated from the assigned residential zip code of the patient to the closest treatment center. County-level social determinants of health were obtained from the AHRF. Individuals were assigned to a county based on the provider 5-digit zip code, which served as a proxy for patient residence. The following social determinants of health variables were used in the analysis: percentage graduated high school, unemployment rate, poverty rate, population density, PCPs per 100,000 population, mental health providers (psychiatrists, community mental health centers, etc) per 100,000 population, median household income, and urban/rural status. Racial and ethnic distributions (deemed surrogate measures of inequality) were the percent of a county residents identified as non-Hispanic White, non-Hispanic Black, Hispanic, Asian, American Indian, Alaska native, native Hawaiian, other Pacific Islander (AIAN NHOPI), or multi-race.

### 2.6. Statistical analysis

Demographic, comorbidity, and health care service utilization characteristics were reported using means and standard deviations (SDs) for continuous variables and frequencies and proportions for categorical variables. The association between baseline characteristics and ESK use was tested using chi-squared tests for categorical variables*, t* tests for normally distributed continuous variables, and Wilcoxon Mann–Whitney *U* tests for non-normally distributed continuous variables.

To assess the association between distance and SDoH on treatment initiation, a parsimonious multivariate logistic regression model with the outcome of ESK initiation (yes/no) was built, using distance to certified treatment center along with SDoH variables as the primary independent variables. The model included the above variables in the full model and the final, parsimonious model was derived from backward elimination with alpha threshold set at 0.05.

The association between distance and SDoH with completion of the induction phase was assessed by logistic regression among individuals who initiated treatment. The full model included distance to certified treatment center, along with age, gender, racial and ethnic distributions, insurance type, selected psychiatric comorbidities, CCI, psychiatry visits and psychiatric prescription medication use in baseline, and SDoH variables. The final model was derived from backward elimination with alpha threshold set at 0.05. To assess the association between distance and SDoH on treatment discontinuation, a parsimonious multivariate Cox proportional hazard model with the outcome of time on treatment (in days) and a discontinuation indicator was built and applied to the population with probable TRD (i.e., those without an MDSI diagnosis). Cox models adjusted for the same covariates as described above. The proportional hazards assumption was assessed using log-log plots. All analyses were conducted using SAS enterprise, version 9.4 (SAS Institute, Cary, NC) and R version 4.1.1 (The R Foundation).

## 3. Results

The study included 308 ESK initiators and 1540 controls. There were no statistically significant differences between ESK initiators and controls on any individual characteristics used to derive the propensity score and standardized mean differences were < 0.09 on all characteristics. ESK initiators had mean age of 48.8 years (SD: 16.9 years), were predominantly female (61.4%) and commercially insured (57.8%; Table 1). The most common psychiatric comorbidities were generalized anxiety disorder (31.5%), sleep-wake disorders (23.1%), other anxiety disorders (17.5%), substance use disorder (11.4%), alcohol use disorder (7.1%), and dysthymic disorder (5.5%). In the year prior to index, most (67.2%) individuals had ≥ 1 visits with a psychiatrist, and 35.4% had ≥ 1 psychotherapy sessions.

**Table 1:** Distribution of demographic and SDoH characteristics among the 308 ESK initiators.

		ESK initiators	
		n	%
Sex	Female	189	61.4%
	Male	119	38.6%
Age (mean, SD)*		(48.8)	(16.9)
Insurance	Commercial	178	57.8%
	Medicare	81	26.3%
	Medicaid	39	12.7%
	VA/Other	10	3.2%
Comorbidities	GAD	97	31.5%
	Sleep-wake disorders	71	23.1%
	Anxiety	54	17.5%
	Hypertension	35	11.4%
	Substance use/addiction disorders	35	11.4%
	Hyperlipidemia	29	9.4%
	Obesity	28	9.1%
	Dysthymic disorder	17	5.5%
Charlson comorbidity index	0	262	85.1%
	1+	46	14.9%
Service utilization in baseline	PCP Visits > Median	113	36.7%
	Any hospital based OP visit	105	34.1%
	Any ED	79	25.6%
	Any IP	53	17.2%
	Any psychotherapy	109	35.4%
	Any psychiatrist	207	67.2%
	Rx fills > median	79	25.6%
	Psychiatric Rx fills > median	90	29.2%
	Any psychiatric office visit	247	80.2%

ED = emergency department, ESK = esketamine, GAD = general anxiety disorder, IP = inpatient, OP = outpatient, PCP = primary care provider, SD = standard deviation, SDoH = social determinants of health.

ESK initiators traveled an average of 7.0 miles (SD: 9.4; median: 4.1 miles) and a total of 99 (32.1%) ESK users did not go to the closest treatment center, traveling an average of 4.8 additional miles to receive treatment.

In comparison, controls lived an average of 14.2 miles to the nearest treatment center (SD: 15.3 miles; median: 7.7 miles; *P* < .01). Controls differed from ESK initiators on the following SDoH measures based on region of residence, residing in areas with lower population density per square mile (1712.4 vs 1886.8; *P* < .01), lower median annual household income ($68,212 vs $74,139; *P* < .01), lower unemployment rate (7.9% vs 8.6%; *P* < .01), higher percent non-Hispanic White population (65.3% vs 59.4%; *P* < .01), lower Asian population (5.3% vs 8.8%; *P* < .01), and a lower AIAN NHOPI population (1.1% vs 1.3%; *P* < .01; Table 2). Controls also resided in areas that were more likely designated as rural (44.9% vs 24.0%; *P* < .01), less likely designated as metropolitan (55.1% vs 76.0%; *P* < .01), and with fewer PCPs(94.5 vs 108.9; *P* < .01) and mental health providers (14.6 vs 19.2; *P* < .01) per capita.

**Table 2:** Distribution of SDoH characteristics and distance to treatment center among ESK initiators and controls.

		Controls		ESK Initiators		*P* value
		1540		308		
		n	%	n	%	
Social Determinants						
	Population density per Sq mile (Mean, SD)	1712.4	5856.8	1886.8	4086.3	<.01
	High school graduation, % (Mean, SD)	89.2	4.4	89.5	4.2	NS
	Median household income, $ (Mean, SD)	68212	17013	74139	19543	<.01
	Mental health access (Providers per 100k)	14.6	13.4	19.2	12.3	<.01
	Primary care provider access (Providers per 100k)	94.5	40.8	108.9	37.4	<.01
	Poverty rate	12.0	4.1	11.8	4.2	NS
	Unemployment rate	7.9	2.1	8.6	2.2	<.01
	Non-Hispanic, white	65.3	19.9	59.4	18.3	<.01
	Non-Hispanic, black	12.6	12.0	13.9	14.5	NS
	Asian	5.3	5.7	8.8	8.2	<.01
	AIAN NHOPI	1.1	1.1	1.3	1.0	<.01
	Hispanic	14.1	14.2	14.8	13.5	NS
	Metro (1 + million population)	849	55.1%	234	76.0%	<.01
	Rural	691	44.9%	74	24.0%	<.01
Distance (miles) to closest treatment center (mean, SD)		(14.2)	(15.3)	(6.9)	(9.4)	<.01

AIAN NHOPI = American Indian, Alaska native, native Hawaiian, other Pacific Islander, ESK = esketamine, SD = standard deviation, SDoH = social determinants of health.

### 3.1. Initiation

**Figure 1.**
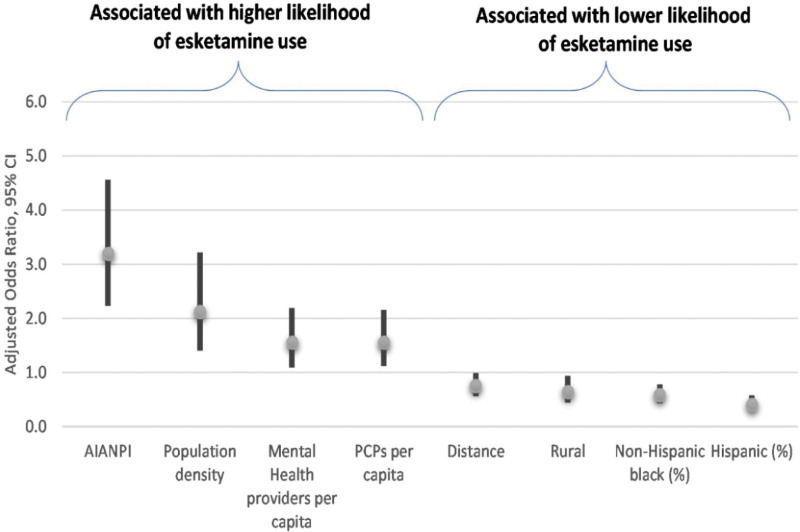
Adjusted odds ratios and 95% confidence intervals among factors significantly associated with initiating ESK treatment. ESK = esketamine.

After adjustment, living > 7.2 miles (the top tertile for distance traveled) away from the closest treatment center was negatively associated with likelihood of initiation (odds ratio [OR]: 0.75; 95% CI: 0.56–0.99). Other factors associated with lower likelihood of ESK initiation were the percent of the population non-Hispanic African American (OR: 0.58; 95% CI: 0.43–0.78), percent of the population Hispanic (OR: 0.40; 95% CI: 0.28–0.58) and living in rural areas (OR: 0.64; 95% CI: 0.44–0.94). Factors associated with higher likelihood of ESK initiation were higher population density (OR: 2.12; 95% CI: 1.40–3.22), percent of population AIAN NHOPI (OR: 3.19; 95% CI: 2.23–4.56), mental health providers per 100,000 population (OR: 1.55; 95% CI: 1.09–2.19), and PCPs per 100,000 population (OR: 1.55; 95% CI: 1.12–2.16; Fig. 1).

### 3.2. Induction

Among the 308 ESK initiators, 133 (43.2%) completed the induction phase. In the unadjusted comparison, compared with individuals who did not complete the induction phase, individuals who completed the induction phase were more likely to reside in metropolitan (82.7% vs 70.3%) areas (*P* < .05), less likely to have comorbid substance use disorder (5.3% vs 16%; *P* < .01), lived in counties with higher high school graduation rate (90.2% vs 89.0%; *P* < .05), more mental health providers per 100,000 population (21.4 vs 17.6; *P* < .01), more PCPs per 100,000 population (116.8 vs 102.9; *P* < .01), lower percent Hispanic population (11.9% vs 17.0%; *P* < .05), and lower CCI score (0.12 vs 0.39; *P* < .01).

**Figure 2.**
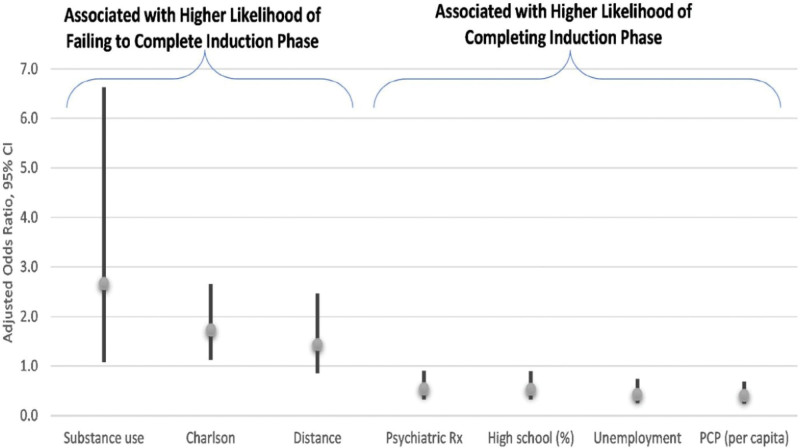
Adjusted odds ratios and 95% confidence intervals among factors associated with completing the ESK induction phase. ESK = esketamine.

After adjustment, substance use disorder (OR: 2.67; 95% CI: 1.08–6.63) and CCI (OR: 1.73; 95% CI: 1.12–2.66) were positively associated with not completing the induction phase. In contrast, baseline history of psychiatric medication use (OR: 0.54; 95% CI: 0.32–0.90), and residing in counties above the median level of high school graduation rate (OR: 0.53; 95% CI: 0.32–0.89), unemployment rate (OR: 0.43; 95% CI: 0.25–0.74), and PCPs per 100,000 (OR: 0.41; 95% CI: 0.24–0.69), were negatively associated with not completing the induction phase (Fig. 2).

### 3.3. Discontinuation

A total of 275 ESK users were not classified as having MDSI and thus were appropriate to transition from the induction phase to a maintenance phase of treatment. Of these, individuals who traveled > 7.2 miles (the top tertile for distance traveled) were significantly less likely to remain on therapy (25.2% vs 36.8%; *P* = .02) through the 6-month follow-up period, with the most difference in discontinuation occurring after the first 25 days (see Figure S2, Supplemental Digital Content, http://links.lww.com/MD/I443, proportion of patients on treatment by days since treatment initiation).

In the unadjusted comparison, individuals were more likely to remain persistent if they were insured commercially (40.0%) compared to Medicare (30.1%) or Medicaid (15.6%) (*P* < .05), or if they resided in counties with higher high school graduation rates (90.3% vs 89.1%; *P* < .05), more mental health care providers per 100,000 population (21.9 vs 18.2; *P* < .01), more PCPs per 100,000 population (117.8 vs 105.1; *P* < .01), lower percent Hispanic population (11.2% vs 16.5%; *P* < .05), or lower percent AIAN NHOPI population (1.1% vs 1.3%; *P* < .05).

**Figure 3.**
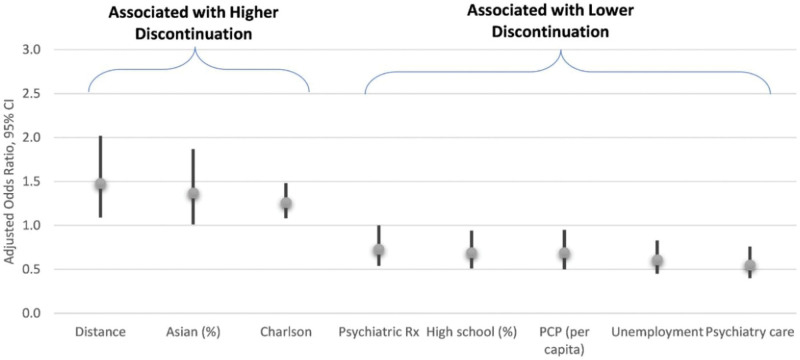
Adjusted odds ratios and 95% confidence intervals among factors associated with discontinuation.

After adjustment, longer distance was positively associated with the discontinuation rate (hazard ratio [HR]: 1.48; 95% CI: 1.09–2.02), as was percent Asian (HR: 1.37; 95% CI: 1.01–1.87), and CCI (1.26; 95% CI: 1.08–1.48), whereas higher unemployment rate (HR: 0.61, 95% CI: 0.45–0.83), higher high school graduation rate (HR: 0.69; 95% CI: 0.51–0.94), higher PCPs per 100,000 population (HR: 0.69, 95% CI: 0.50–0.95), baseline psychiatry visits (HR: 0.55; 95% CI: 0.40–0.76), and baseline psychiatric medication use (HR: 0.73; 95% CI: 0.54–1.00), were associated with greater persistence (Fig. 3).

## 4. Discussion

Our results contribute to the growing body of evidence that racial and ethnic inequality, SDoH, and distance to be traveled may represent barriers to access that adversely affect utilization of mental health care services. Our study expands upon prior research by assessing 3 specific aspects of treatment (initiation, completing a clinically important induction phase, and early discontinuation), and by studying a population with challenging-to-treat MDD that has previously sought care and been referred to a certified treatment center.

The SDoH measured in this study can be categorized into race and ethnicity (surrogate measures of inequality), socioeconomic status (household income, education, and unemployment), and urbanicity, as well as the built environment (PCP and mental health providers per capita, population density, and urbanicity). In our study, characteristics within each domain were independently associated with ESK utilization.

Indicators of race and ethnicity were associated with both treatment initiation and early treatment discontinuation. Individuals who reside in communities with higher rates of non-Hispanic Black or Hispanic populations were each about half as likely to initiate ESK treatment, while early treatment discontinuation was 37% higher in Asian communities. Though these associations don’t provide insight into causality, perceived need for mental health care varies dramatically across racial groups and ethnicities,^[24]^ as do reasons reported for not seeking treatment and for dropping out of treatment.^[25]^ These differences remain across all levels of severity^[24]^ and persist after accounting for differences in mental health distress and impairment.^[26]^ Asian Americans generally underutilize mental health services.^[27,28]^ The elevated odds of discontinuation among individuals who reside in Asian communities align with results from a national population-based survey in which Asian respondents reported significantly lower odds of completion ≥ 8 visits with a psychiatrist.^[29]^

The built environment – broadly defined as the physical infrastructure and design of an area in which individuals live and work – has numerous definitions,^[30]^ but regardless of definition, impacts individuals’ mental health.^[31,32]^ In our study, the built environment is measured broadly by population density, by urbanicity, and, more specifically, by the availability of mental health care services, which vary dramatically by geography and by the socioeconomic status of communities. No characteristics of the built environment were associated with completing induction or early discontinuation. However, higher population density, PCPs per capita, and mental health care providers per capita were all associated with greater likelihood of initiation.

In our study, distance was associated with a 25% lower likelihood of initiation and with nearly a 2-fold increase in the likelihood of discontinuing treatment early in the therapeutic course. Greater travel distance has been reported to reduce utilization of mental health care services among older adults with serious mental illness,^[33]^ among a population-based sample of individuals who initiated antidepressant therapy,^[34]^ and among a pediatric population with depression.^[35]^ Distance has been reported to negatively impact overall utilization of mental health care services, treatment initiation rates,^[35]^ and treatment completion rates.^[35]^ Evidence also suggests socioeconomically disadvantaged individuals are significantly more sensitive to distance than those with more economic resources. Consistent with these results, socioeconomically disadvantaged individuals are more likely to report transportation as a barrier to mental health care.^[17]^

The implications of available socioeconomic resources on mental health care utilization are referenced throughout this discussion. Individuals who reside in low-income areas may struggle with transportation and available time, or reside in areas with fundamentally fewer mental health care resources. In a US national study of 31,836 zip code areas, Cummings et al^[36]^ reported that office-based mental health professionals were more likely located in higher socioeconomic status areas and that, in contrast, mental health treatment facilities were more likely to be located in lower socioeconomic status areas.

### 4.1. Consequences and implications

Our results should be interpreted within the population context. TRD and MDSI are significant clinical and public health issues. In the United States, an estimated 10% of adults meet diagnostic criteria for MDD annually and of these, an estimated 30% meet criteria for TRD^[9]^ and more than 1 in 5 contemplate suicide.^[37]^ Further, more than half of the US population is exposed to ≥ 1 SDoH.^[38]^ Finally, the associations between treatment continuation, SDoH, and distance are noted among individuals who have already sought mental health care, exhibiting an openness to treatment and often having already attempted numerous therapeutic interventions. In light of these facts, our results are potentially more important.

## 5. Limitations

The study is subject to several limitations. First, the analytical dataset does not include eligibility and so complete capture of all health care utilization cannot be verified. Second, mental health diagnoses may be underreported. Given that mental health diagnoses such as suicidal ideation and attempt are used to assign an MDSI diagnosis, underreporting may lead to selection bias where selected patients may be different from those that are not selected due to underreporting. Third, the distance measure is not a precise measure of either distance traveled or the time required to travel to a treatment center. As the source dataset is Health Insurance Portability and Accountability Act-compliant, individuals were assigned to the centroid of the assigned 5-digit zip code, which in turn was assigned based on an algorithm of health care provider addresses. Finally, social determinants were not defined at the individual but rather county of residence level. AHRF social determinants and health access data are routinely used in population-based research but lack the precision to report on nuances in relationships between social determinants and outcomes, as illustrated in a study by Sorkin et al^[39]^ that demonstrated substantial variation in barriers to mental health care across Asian subgroups.

## 6. Conclusions

These research insights shed additional light on the influence of racial and ethnic inequality, social determinants, and distance as factors that contribute to the unmet treatment needs of individuals with TRD or MDSI^[40]^ and to the delivery of guideline-aligned mental health care.^[41]^ Mental health providers who refer patients for ESK treatment and the providers associated with certified treatment centers may need to consider social determinants and distance to treatment center when providing scheduling and support services. Health care providers should consider barriers and tailor patient engagement strategies to improve access to appropriate treatment. Alternative delivery models may have the potential to improve access for patients who may encounter access barriers.

## Acknowledgment

The authors would like to thank Margaret L. Stinstrom for administrative support.

## Author contributions

**Conceptualization:** Joshua Liberman, Jacqueline Pesa, Kruti Joshi, Lisa Harding.

**Data curation:** Pinyao Rui.

**Formal analysis:** Pinyao Rui.

**Funding acquisition:** Jacqueline Pesa.

**Methodology:** Joshua Liberman, Jacqueline Pesa, Pinyao Rui.

**Project administration:** Joshua Liberman, Jacqueline Pesa, Pinyao Rui, Kruti Joshi.

**Writing – original draft:** Joshua Liberman.

**Writing – review & editing:** Joshua Liberman, Jacqueline Pesa, Pinyao Rui, Kruti Joshi, Lisa Harding.

## Supplementary Material
