# Supplementary material for: Social determinants and distance from certified treatment centers are associated with initiation of esketamine nasal spray among patients with challenging-to-treat major depressive disorder
Source: Medicine (Baltimore). 2023 Feb 17;102(7):e32895. doi: 10.1097/MD.0000000000032895 (PMC9935983; doi:10.1097/MD.0000000000032895)
Supplement: Supplementary file 1 [file medi-102-e32895-s001.pdf]

Supplemental Table 1.

Suicidal ideation will be defined by a claim associated with an ICD-10 code of R45.581.

Suicidal behavior will be defined as a suicide attempt or an attempt at self-harm, as defined below.

<u>Suicide Attempt:</u> T14.91

<u>Intentional self-harm by poisoning (Hedegaard et al. 2018):</u> T36.[0-9]X2*, T37.[0-9]X2*, T38.[0-7]X2*, T38.8[0-9]2*, T38.9[0-9]2*, T39.0[1-9]2*, T39.1X2*, T39.2X2*, T39.3[1-9]2*, T39.4X2*, T39.8X2, T39.92X*, T40.0[0-5]X2*, T40.6[0-9]2*, T40.7X2*, T40.8X2*, T40.902*, T40.992*, T41.0X2*, T41.1X2*, T41.2[0-9]2*, T41.3X2*, T41.42X*, T41.5X2*, T42.[0-6]X2*, T42.72X*, T42.8X2*, T43.0[1-2]2*, T43.1X2*, T43.2[0-9]2*, T43.[3-4]X2*, T43.5[0-9]2*, T43.6[0-9]2*, T43.8X2*, T43.92X*, T44.[0-8]X2*, T44.9[0-9]2*, T45.[0-4]X2*, T45.5[1-2]2*, T45.6[0-9]2*, T45.7X2*, T45.8X2*, T45.92X*, T46.[0-8]X2*, T46.9[0-9]2*, T47.[0-8]X2*, T47.92X*, T48.[0-1]X2*, T48.2[0-9]2*, T48.[3-6]X2*, T48.9[0-9]2*, T49.[0-8]X2*, T49.82X*, T50.[0-8]X2*, T50.9[0-9]2*, T50.A[1,2,9]2*, T50.B[1,9]2*, T50.Z[1,9]2*

<u>Intentional self-harm by toxic effect:</u> T51.[0-8]X2*, T51.92X*, T52.[0-8]X2*, T52.92X*, T53.[0-7]X2*, T53.92X*, T54.[0-3]X2*, T54.92X*, T55.[0-1]X2*, T56.[0-7]X2*, T56.8[1,9]2*, T56.92X*, T57.[0-8]X2*, T57.92X*, T58.[0,2]2X*, T58.2X2*, T58.8X2*, T58.92X*, T59.[0-7]X2*, T59.8[1,9]2*, T59.92X*, T60.[0-8]X2*, T60.92X*, T61.[0,1]2X*, T61.7[7,8]2*, T61.8X2*, T61.92X*, T62.[0-2,8]X2*, T62.92X*, T63.0[0-4,6-9]2*, T63.1[1,2,9]2*, T63.2X2*, T63.3[0-3,9]2*, T63.4[1-6,8]2*, T63.5[1,9]2*, T63.6[1-3,9]2*, T63.7[1,9]2*, T63.8[1-3,9]2*, T63.92X*, T64.[0,8]2X*, T65.0X2*, T65.1X2*, T65.2[1,2,9]2*, T65.[3-6]X2*, T65.8[1-3,9]2*, T65.92X*

<u>Asphyxiation:</u> T71.1[1-3,5,6,9]2*, T71.2[2,3]2*

<u>Intentional self-harm by drowning:</u> X71.[0-3,8,9]XX*,

<u>Intentional self-harm by gun:</u> X72.XXX*, X73.[0-2,8,9]XX*, X74.0[1,2,9]X*, X74.8XX*, X74.9XX*

<u>Other intentional self-harm:</u>X7[5-6].XXX*, X77.[0-3,8-9]XX*, X78.[0-2,8-9]XX*, X79.XXX*, X80.XXX*, X81.[0-1,8]XX*, X82.[0-2,8]XX*, X83.[0-2,8]XX*
