# Supplementary figures and images for: Social determinants and distance from certified treatment centers are associated with initiation of esketamine nasal spray among patients with challenging-to-treat major depressive disorder
Source: Medicine (Baltimore). 2023 Feb 17;102(7):e32895. doi: 10.1097/MD.0000000000032895 (PMC9935983; doi:10.1097/MD.0000000000032895)

Supplemental Figure 2.

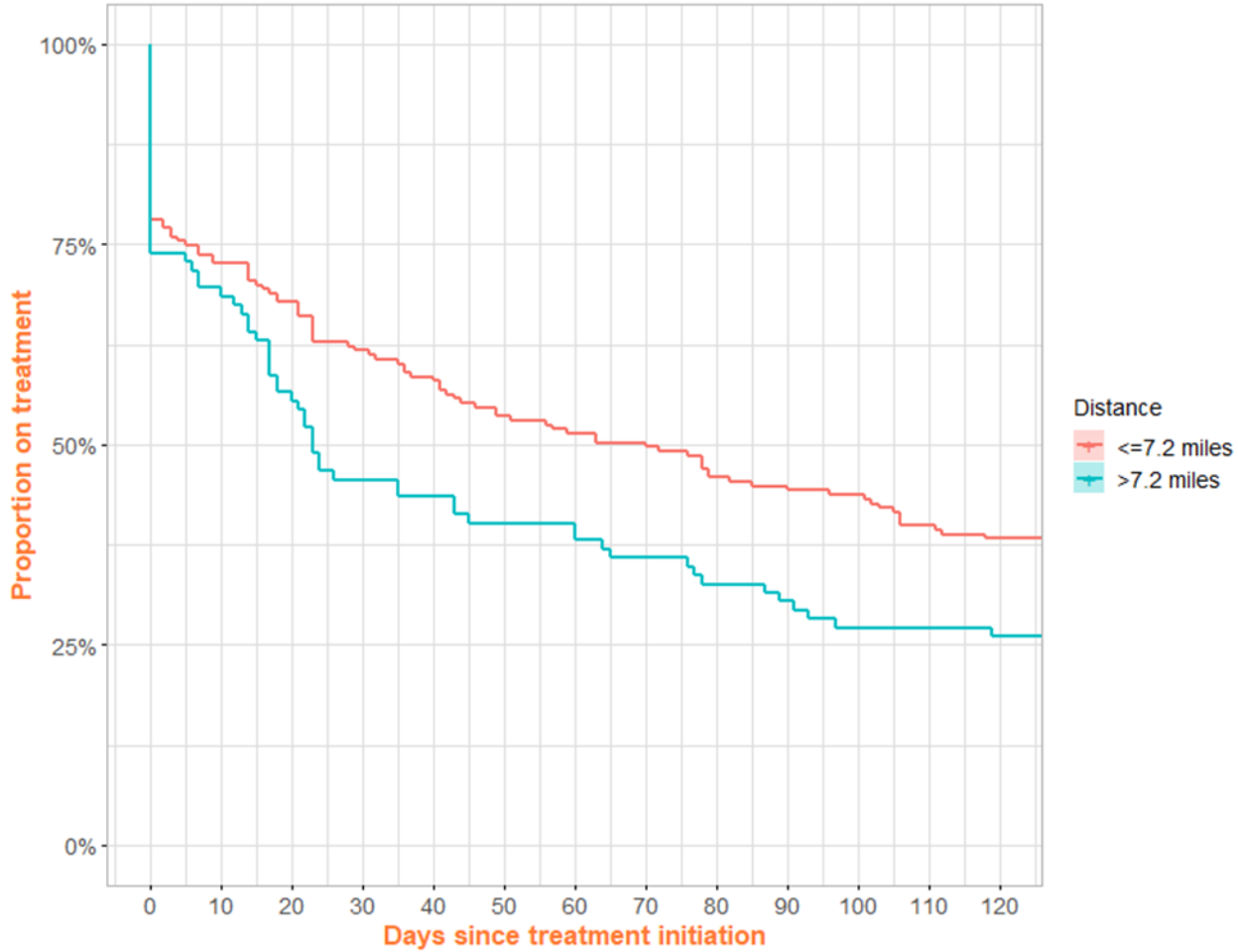

Supplement: Supplementary file 3 [file medi-102-e32895-s003.pdf]
